# Inspiratory Muscle Training for Obstructive Sleep Apnea: Protocol Development and Feasibility of Home Practice by Sedentary Adults

**DOI:** 10.3389/fphys.2021.737493

**Published:** 2021-11-04

**Authors:** Beatrix Krause-Sorio, Eunjoo An, Andrea P. Aguila, Fernando Martinez, Ravi S. Aysola, Paul M. Macey

**Affiliations:** ^1^UCLA School of Nursing, University of California, Los Angeles, Los Angeles, CA, United States; ^2^Division of Pulmonary and Critical Care, David Geffen School of Medicine, University of California, Los Angeles, Los Angeles, CA, United States

**Keywords:** sleep apnea, breathing, sleep, training, intervention, inspiratory muscle training, respiration, strength

## Abstract

**Background:** Inspiratory muscle training (IMT) may improve respiratory and cardiovascular functions in obstructive sleep apnea (OSA) and is a potential alternative or adjunct treatment to continuous positive airway pressure (CPAP). IMT protocols were originally designed for athletes, however, we found some OSA patients could not perform the exercise, so we aimed for a more OSA-friendly protocol. Our feasibility criteria included (1) participants successfully managing the technique at home; (2) participants completing daily practice sessions and recording data logs; and (3) capturing performance plateaus to determine an optimal length of the intervention.

**Methods:** Five sedentary OSA patients participated in this feasibility study (three men, mean age = 61.6 years, SD = 10.2). Using a digital POWERbreathe K4 or K5 device, participants performed 30 daily inhalations against a resistance set at a percentage of maximum, recalculated weekly. Participants were willing to perform one but not two daily practice sessions. Intervention parameters from common IMT protocols were adapted according to ability and subjective feedback. Some were unable to perform the typically used 75% of maximum inspiratory resistance so we lowered the target to 65%. The technique required some practice; therefore, we introduced a practice week with a 50% target. After an initial 8 weeks, the intervention was open-ended and training continued until all participants demonstrated at least one plateau of inspiratory strength (2 weeks without strength gain). Weekly email and phone reminders ensured that participants completed all daily sessions and logged data in their online surveys. Weekly measures of inspiratory resistance, strength, volume, and flow were recorded.

**Results:** Participants successfully completed the practice and subsequent 65% IMT resistance targets daily for 13 weeks. Inspiratory strength gains showed plateaus in all subjects by the end of 10 weeks of training, suggesting 12 weeks plus practice would be sufficient to achieve and capture maximum gains. Participants reported no adverse effects.

**Conclusion:** We developed and tested a 13-week IMT protocol in a small group of sedentary, untreated OSA patients. Relative to other IMT protocols, we successfully implemented reduced performance requirements, a practice week, and an extended timeframe. This feasibility study provides the basis for a protocol for clinical trials on IMT in OSA.

## Introduction

Over 10% of the population suffers from obstructive sleep apnea (OSA), a disorder characterized by repeated pauses in breathing during sleep due to collapsing of the upper airway (Peppard et al., 2013; Marshall et al., 2014). The condition is a major risk factor for cardiovascular disease including hypertension and cardiovascular disease (Monahan et al., 2009; Peppard et al., 2013; Javaheri et al., 2017; Whelton et al., 2018). Nightly use of continuous positive airway pressure (CPAP), the standard treatment for OSA, resolves the breathing disruptions and improves some of the symptoms, but shows mixed results for reducing blood pressure (BP) (Barbé et al., 2012; McEvoy et al., 2016; Whelton et al., 2018). Furthermore, CPAP adherence is often low, as patients experience it as intrusive and difficult to wear throughout the night. In some patients, weight loss and physical exercise improve daytime symptoms and breathing during sleep, but as with other chronic conditions, more often than not, stable long-term health behavior change in OSA is not achieved (Aiello et al., 2016; Vranish and Bailey, 2016; Souza et al., 2018; Carneiro-Barrera et al., 2019). Consequently, there remains a need for complementary or alternative interventions for treating OSA and its comorbidities.

One potentially beneficial intervention for people with OSA is inspiratory muscle training (IMT). IMT is the practice of strengthening respiratory muscles involved in inhalations (Larson et al., 1988). Simplistically, OSA arises from a failure of breathing, therefore training breathing may improve symptoms. More specifically, since OSA involves the collapse of the upper airways with inspiration during sleep, IMT may reduce the number and/or severity of apneas by improving upper airway muscle tone (How et al., 2007). In addition, IMT may also improve cardiovascular symptoms, since studies in normotensive adults and patients with OSA found significant reductions in BP with IMT, and improved functional capacity in people with heart failure (Ferreira et al., 2013; Vranish and Bailey, 2015, 2016; Posser et al., 2016; DeLucia et al., 2018; Fernandez-Rubio et al., 2020; Ramos-Barrera et al., 2020). The mechanism of IMT effects on cardiovascular function is unknown, but may be due to either associations between respiratory and cardiovascular activity, or due to potential positive IMT-related effects on stress (Grossman et al., 2001; HajGhanbari et al., 2013; de Abreu et al., 2017; Fernandez-Rubio et al., 2020). Regardless of the mechanism, the evidence suggests that IMT has the potential to improve both breathing and cardiovascular symptoms in OSA.

Inspiratory muscle training can be performed systematically using devices that provide quantifiable resistance targets and instant performance feedback. These targets are usually set as a percentage of the maximum inspiratory pressure that an individual can generate, as measured by a sensor in the device. The technique is akin to weight training where a set of repetitions is performed using a fixed weight. As the respiratory muscles gain strength, the IMT device allows the target resistance to be increased, so the training adapts to the person’s current strength. A common target resistance in IMT studies is 75% of the maximum capacity, in a set of 30 repetitions performed twice daily for 6 weeks (HajGhanbari et al., 2013). However, such parameters are not achievable by all non-athletes or people with other chronic conditions who may have limited fitness or mobility. Therefore, modifications are needed for such populations. For example, in IMT studies with older adults, parameters vary from 4 to 8 weeks of training duration, 30–80% target resistance, and between 5 and 7 weekly sessions (Seixas et al., 2020). Although one research group developed and successfully tested a protocol for people with OSA for 6 weeks, 75% of maximum target resistance performed twice daily (Vranish and Bailey, 2016; Ramos-Barrera et al., 2020), several of our participants required repeated practice sessions across several days to learn the technique, and were still unable to inhale against the 75% target resistance. Hence, they were unable to complete the exercise. Other participants were reluctant to practice two times a day. We speculate that compared to earlier studies, our participants were sedentary with reduced capacity to perform the resistance training, and that they had less time and motivation to practice twice daily, since they were not retired. To develop a protocol that was suited to our sedentary patient population, we aimed to modify the previously tested parameters, such that participants could successfully perform the practice and adhere to the training schedule. Our objective was to develop a clinical trial IMT protocol for untreated, sedentary OSA patients. Our approach was to work with a small group of sedentary OSA patients to test and adapt previously tested IMT parameters based on subjective feedback and quantitative measures. We also aimed to derive an empirical basis for a clinical trial duration. Therefore, the trial began with an 8-week minimum duration and continued until performance plateaued or until participants reported unwillingness to continue.

Our feasibility criteria for the protocol included:

(1) All participants had to be able to successfully perform the practice using the same settings (e.g., starting resistance);
(2) Participants had to complete daily practice sessions;
(3) Participants had to complete the protocol until the end; and
(4) Participants had to show sustained increases and eventually plateaus in inspiratory strength.

## Methods

### Participants

We recruited participants at the University of California, Los Angeles (UCLA) campus and the local community *via* digital and printed fliers. Inclusion criteria were a diagnosis of mild, moderate, or severe OSA based on a two-night home sleep study using a polysomnography device scored according to the 2012 American Academy of Sleep Medicine criteria (Ayappa et al., 2008; Berry et al., 2012); aged 21–75; and no current or previous sustained use of OSA treatment. Exclusion criteria included a history of stroke, heart failure, or other major cardiovascular disease, a history of diagnosed mental health conditions other than unipolar depression or anxiety disorder, respiratory illness other than OSA (including contraindications for IMT such as pulmonary hypertension), cystic fibrosis, presence of mass brain lesions, renal failure (requiring dialysis) and drug abuse. While IMT is considered a low risk practice, the resulting large negative pressure swings within the chest (intra-thoracic decompression) hold a potential risk of sub-atmospheric pressures in the chest, throat, inner ear, and sinuses (McConnell et al., 2005). Therefore, exclusion criteria due to IMT contraindications also included a history of spontaneous pneumothorax, traumatic pneumothorax that was not fully healed at the time of recruitment, burst eardrum or other conditions of the eardrum, including recent eardrum surgery, unstable asthma, and abnormally low perception of dyspnea.

The procedures were approved by the UCLA Institutional Review Board. Participants provided written informed consent signed digitally for initial remote activities and in written form at the first in-person visit. There was no public involvement as this was an early-stage study.

### Screening

Participants initiated contact by phone, email, or text, whereupon a researcher contacted them by phone. The telephone screening involved assessing potential participants’ medical history, including the assessment of the previously listed inclusion and exclusion criteria. Potential participants then completed an online survey assessing further details about medical history, demographics, sleeping times, subjective sleep quality, and daytime sleepiness. Participants who met the study criteria were subsequently referred for a home sleep study.

### Home Sleep Study

Home sleep studies were executed by a third party, SleepMed (SleepMed Inc., Peabody, MA, United States), in conjunction with the UCLA Sleep Disorders Center. Within 48 h of enrollment into the study, participants received a phone call by a SleepMed representative to schedule an appointment for delivery. Upon receiving the portable SleepMed polysomnography device [ARES^TM^ Home Sleep Test system (Ayappa et al., 2008)] in the mail, participants wore the device strapped to their foreheads for two consecutive nights with a nightly minimum of 4 h. The device recorded heart rate, breathing, oxygen saturation, snoring from auditory recordings, and electroencephalogram (EEG) during their sleep. The return packaging and paid postage was included in the original shipment. After completion of the second night, participants returned the device to SleepMed by mail, where the data was uploaded and scored by a sleep technologist in a draft report. This report was made available to the research group and interpreted by the sleep physician of our research group. The apnea hypopnea index (AHI) is the sum of apneas and hypopneas during sleep per hour and was derived from the SleepMed data. Mild sleep apnea is reflected by 5–15 events per hour, moderate by 15–30 and severe by over 30 events per hour. The Respiratory Disturbance Index (RDI) was computed from the number of apneas per hour, the number of hypopneas per hour, and the number of respiratory effort-related arousals (RERAs) per hour during sleep. Mild OSA is indicated by an RDI score from 5 to 15, moderate 15 to 30, and severe over 30. An OSA diagnosis was provided within 2 weeks of the data upload. Participants subsequently received their sleep report *via* a secure and password protected system from the research group. If a diagnosis of OSA was made, participants were eligible for the study.

### Inspiratory Muscle Training

Inspiratory muscle training is performed by inhaling against a resistance a given number of times. For the purposes of controlling the intervention parameters and verifying the number of repetitions, we used a device that would provide a consistent, fixed resistance. POWERbreathe (POWERbreathe International Ltd., Southam, United Kingdom) provides such palm-sized, handheld devices for IMT. Previous studies have used the K3 model (Vranish and Bailey, 2016), but upon testing this model, we were unable to manually adjust the resistance settings, therefore we selected models K4 and K5 instead. Both are digital devices and include software that logs and tracks performance over time. Both models provide immediate feedback to the user. These devices adapt the resistance to match the decline in strength of each individual breath to allow for greater flow and maximum volume. The target resistance was set as a percentage of the maximum inspiratory strength based on the average of three trials. A nose clip was used to prevent nasal breathing. The participant was instructed to stand up with their backs straight so they could maximally fill their lungs and expand their rib cages. The mouthpiece was inserted between the lips upon switching on of the device. Basic instructions were as follows: “*Thirty breaths reaching the target threshold should be completed in the following way: inhalations should be fast, deep and noisy, with the shoulders remaining relaxed. The diaphragm should be expanding, and the lungs should be filled as much as possible. You should feel the deep breaths stretching the rib cage and shoulder muscles. Exhalations should be much slower and quieter than inhalations. In order to prevent lightheadedness, the breath should be held after each exhalation until you feel you need to breathe in again. The IMT device displays a count-down from 30 to 0, and only records inhalations that exceed the target resistance*.” If the participant was unable to reach the target resistance, another trial was added to complete the 30 breaths. Each set required 5–10 min to complete.

### Intervention

Prior to starting the training, a research team member worked with the participants to test and adapt the IMT parameters. Experiences with initial attempts to train participants on the use of the device demonstrated that the maximum starting target resistance settings for each individual participant varied, even though each was calibrated to the maximum strength of the individual. While some participants found it easy to start at 75% resistance or more, some struggled to complete even a few breaths at this level. After discussions and feedback from the participants, we made two adaptations: (1) Participants initially practiced at a lower target resistance with the goal of learning the technique without the performance pressure. Typically, this required them to start with an inhale followed by a full exhale in preparation of the intended forceful inhalation. This allowed them to take a longer than usual sustained inhalation; (2) We set the ongoing target at 65% of the maximum, a level that was achievable for all participants. We found 65% to be the highest resistance that all five participants were able to perform.

We found that some participants had difficulties interacting with the device and required more demonstrations and guidance. Therefore, in addition to the instructions above, we provided in-person training and created a video demonstrating the correct technique that participants could watch at home. Participants were also provided with a paper copy of instructions on usage of the device and proper technique. The resulting protocol and modifications are shown in Table 1. The daily session duration was reported to be initially 10 min, and after a week of practice reduced to typically 5 min.

**TABLE 1:** Protocol modifications after testing five OSA participants.

Component	Standard	Modified	Rationale
Training period	None	1 week at 50% resistance	Training period allowed people to learn the technique correctly. Fifty percent target provides resistance without being overly challenging.
Training materials	None	Online videos and paper handout demonstrating techniques and troubleshooting	Participants were able to address difficulties, especially in the first 2–3 weeks of training, as they were becoming familiar with the technique. These materials improved people’s correct use of the device.
Target resistance	75%	65%	We aimed for consistency across participants. The standard 75% resistance were not attainable by all participants. However, 65% was achievable by all participants. We concluded that sedentary OSA participants may not be able to reach the standard target resistance that is used by people who exercise.
Daily practice sessions	Twice a day (more recent studies once a day)	Once a day	In order to maintain 100% adherence and consistency across participants for the entire training period, we reduced the number of training sessions a day to one. Some participants expressed concerns about practicing twice a day, while none expressed concerns about one session a day.
Duration	6 weeks	13 weeks (1 week 50% and 12 weeks 65%)	We determined the duration based on a plateau of strength increases and participant acceptability.

### Data Acquisition

Data entry of the IMT device data was completed by participants at home *via* an online survey software with encrypted connectivity using their unique study identifier. The variables included inspiratory resistance in percent of the maximum, strength index (cmH_2_O), volume in liters (L) and flow in liters per second (L/s). Measures of interest included performance data from the IMT device (target resistance, volume, and strength index). Data acquired with the polysomnography device during sleep included SaO_2_, which was also used for OSA diagnosis. These sleep study data and reports were available online *via* the SleepMed portal, but only de-identified data were recorded locally by the research team. This included AHI and RDI scores. Other measures of interest acquired from the sleep study and subjective ratings provided during the online survey included self-rated sleep quality ranging from 0 to 10 (poor to good), and excessive daytime sleepiness (ESS) score.

### Analysis

We computed descriptive statistics in Microsoft Excel, including means, medians, standard deviations (SD), and range (minimum to maximum). We used SPSS version 27 to test within-group changes in sleep data, SaO_2_ and IMT device data using the non-parametric Wilcoxon signed-rank test. Non-parametric correlations were tested using Kendall’s tau. In addition, we visualized individual data points for each participant, as well as the group mean and standard deviation. The visualizations aided the detection of the performance increases and plateaus, based on which we adapted the duration of the intervention. We defined a plateau as the absence of an increase in inspiratory strength for three consecutive weeks.

### Protocol Duration

We aimed to derive an empirical basis for trial duration, so the trial duration began open-ended, with participants consenting to participate for up to 8 weeks at a time. We aimed to continue the intervention until we could no longer observe substantial strength increases as measured with the IMT device, or until participants reported unwillingness to continue. Our criterion was to end the protocol after all participants demonstrated a 3-week period with no further increase in strength. This duration differed between participants; therefore, the weakest participants would serve as the criterion.

Based on difficulties of some participants to operate the device and successfully perform the inspirations at the same time, the target resistance was set to 50% for a practice week. This allowed the participants to familiarize themselves with the device use at home without struggling to also complete 30 breaths at a higher resistance. At the beginning of the second week of training, the target resistance was then increased to 65%. At the beginning of each following week, a new maximum strength was determined, and the 65% target re-calculated accordingly. The target could only be increased, not decreased. If a lower maximum strength was measured, the target was kept at the previous level. Therefore, it was important that participants were able to build strength from the beginning without struggling with too high of a resistance. Once a week, the project director reached out to participants by phone to discuss exercise progress and answer questions, record subjective experiences and support protocol adherence.

## Results

**FIGURE 1:**
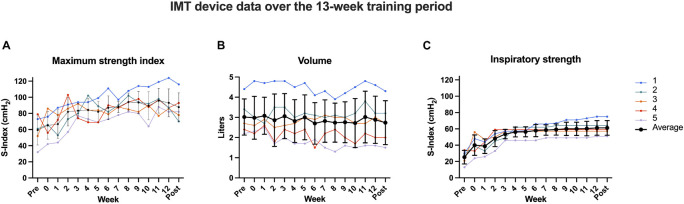
Inspiratory muscle training (IMT) device data over the course of the intervention. Each colored line depicts a different participant, while the thick black line reflects the group average with its standard deviation. Panels: maximum inspiratory strength index **(A)**, respiratory volume in liters **(B)**, and average inspiratory strength **(C)** of five untreated OSA patients from baseline across 13 weeks of IMT. While participant 1 had a higher baseline on all measures and continued to increase both resistance and strength until the end of the trial, the average of the group reached a ceiling effect in strength index after approximately 8 weeks (with slight fluctuations) and after ca. 4 weeks in resistance.

**FIGURE 2:**
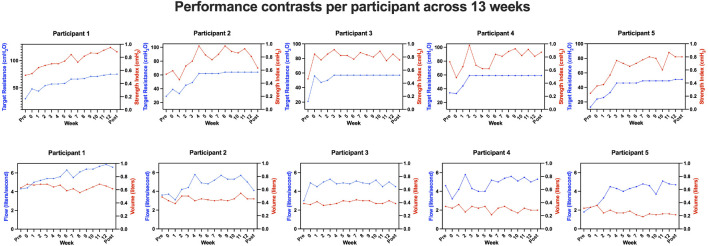
Inspiratory muscle training (IMT) device data for each participant per week. Note the fluctuations in strength index (top panel, right *y*-axis, red) despite the flattening of the curve of the target resistance (top panel, left *y*-axis, blue) for participants 2–5. Only for participant 1, there was a steady increase in both measures over the entire course of the intervention. The pattern of the flow curve (bottom panel, blue) corresponds to the pattern of the strength index (top panel, red). For better visualization of the different scales, both are depicted in separate graphs. The volume curve (bottom panel, red) shows less fluctuations and no overall gain across the training period.

Five sedentary adults with OSA completed the protocol (three men; two women; mean age = 62.2 years, SD = 10.5; mean body-mass index = 33.9, SD = 4.3). All participants reported to be willing and motivated to complete one daily session for the initial 8 weeks. Since our criterion of all participants reaching a plateau had not been met after 8 weeks, all participants were enrolled in a second open-ended period. After 13 weeks, all participants had achieved a plateau. Therefore, we finalized the intervention after 13 weeks. Week 0 reflects the practice week in which participants practiced at 50% maximum resistance, which was a low effort for all five participants. Week one subsequently involved the first week of enhanced effort. Participants then continued IMT until the end of week 13 (see Figure 1). Resistance across the group still increased through weeks 6–8 and appeared to plateau at week 10. Even though resistance stagnated before week 10 for some participants, we still observed significant individual variability in strength until the end of the 13 weeks. Figure 2 depicts each participant’s time course of target resistance relative to strength index (top panel) and volume relative to flow (bottom panel). Technical difficulties were reported and resolved in communication with the team members whenever necessary, but participants reported no side effects or discomfort throughout or after completion of the intervention.

**FIGURE 3:**
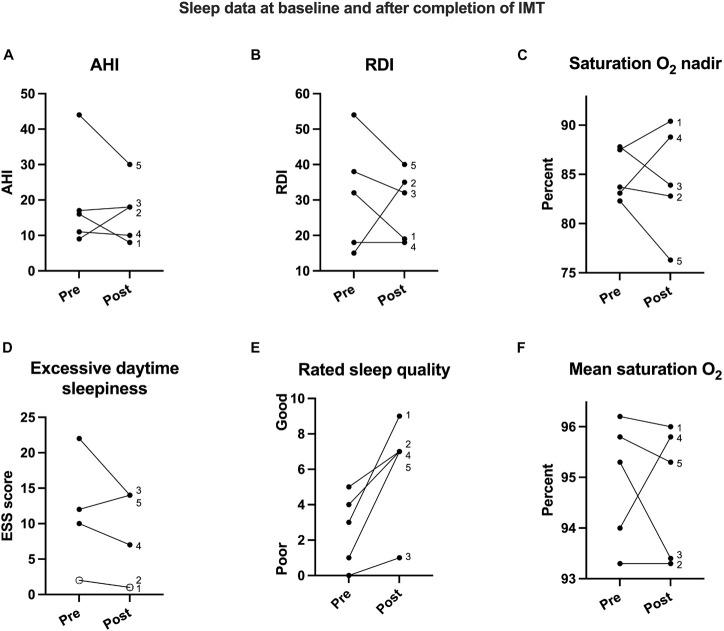
Sleep data for all five participants labeled 1 through 5. While apnea hypopnea index (AHI; **A**), respiratory disturbance index (RDI; **B**), oxygen saturation at nadir (SaO_2_; **C**), and the all-night oxygen saturation average (SaO_2_; **F**) showed individual variation, subjective rating for daytime sleepiness (**D**; participants 1 and 2 overlap here) decreased and therefore improved, and rated sleep quality **(E)** improved in all five participants from baseline to post-IMT intervention.

**FIGURE 4:**
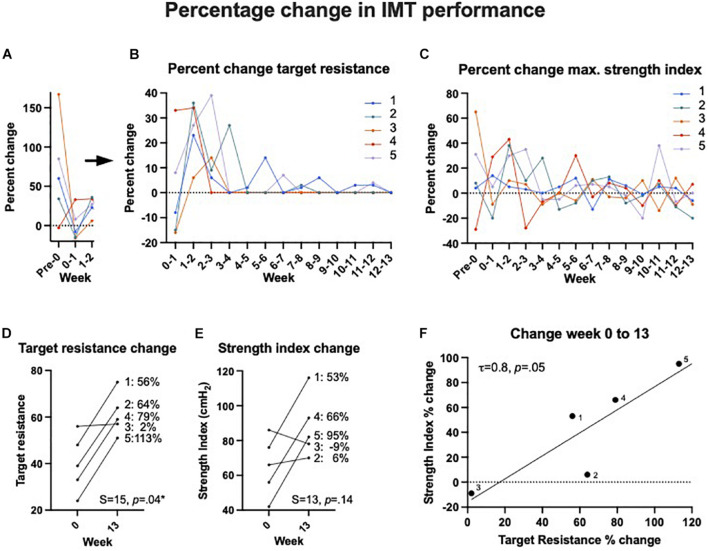
Inspiratory muscle training (IMT) effects on device performance in percentage change. Panels **(A,B)** show the percent change in target resistance measured in cmH_2_O from 1 week to the next. For better visualization and due to the large drop from the first attempt until the end of the practice week (week 0), panel **(A)** shows the data points at a different *y*-axis scale separately from panel **(B)** the following weeks. After 8–9 weeks, there was little to no change in target resistance, i.e., participants were unable to increase their inspiratory strength further at that point. Note that this was not the case for participant 1, whose performance was superior to the other 4. **(C)** The percent change of maximum strength index from 1 week to the next showed greater overall variability but except individual peaks, the general trend evened on the lower end after 4–5 weeks. **(D)** The overall percentage of change in target resistance (measured in cmH_2_O) and **(E)** maximum strength index from week 0 to week 13. **(F)** There was a significant association between target resistance and strength index change in percent from week 0 to week 13 as one would expect.

All baseline and follow-up data are listed in Table 2: there were no significant within-subject changes from baseline to 13-week follow-up for AHI, RDI, ESS, SaO_2_ at nadir, and baseline SaO_2_ (*p*s > 0.59; Figure 3). There was a significant increase in target resistance (cmH_2_O) from week 0 to week 13 (*S* = 15, *p* = 0.04), but not in strength index (*p* = 0.27; see 13-week individual data Figures 4A–C and pre-to-post differences Figures 4D,E). The association between this 12-week increase in percent change target resistance and strength index was marginal (τ = 0.8, *p* = 0.05; Figure 4F). The IMT device data demonstrated that participants gained an average of 15–20% in inspiratory muscle strength over the course of 4 weeks (see Figure 1C).

**TABLE 2:** Descriptive statistics and within-group change of IMT device data from baseline to follow-up.

	Baseline (week 0)		13-week follow-up		Within-group change
	Mean (SD)	Range	Mean (SD)	Range	Wilcoxon signed rank test
Target resistance (cmH_2_O)	40.0 (12.5)	24–56	61.2 (9.0)	51–75	*S* = 15, *p* = 0.04*
Strength index (cmH_2_O)	65.2 (17.2)	42–86	87.8 (17.8)	70–116	*S* = 13, *p* = 0.14
Flow (L/s)	3.7 (1.02)	2.3–4.9	5.0 (1.0)	4.1–6.6	*S* = 13.5, *p* = 0.1
Volume (L)	3.0 (1.1)	2.2–4.8	2.7 (1.1)	1.5–4.3	*S* = 3.5, *p* = 0.28

	**Baseline (pre-practice)**		**13-week follow-up**		

AHI	19.4 (14.2)	9–44	16.8 (8.7)	8–30	*S* = 5.5, *p* = 0.59
RDI	31.4 (15.8)	15–54	28.8 (9.8)	18–40	*S* = 4.0, *p* = 0.72
ESS	9.6 (8.3)	2–22	7.4 (6.5)	1–14	*S* < 0.0001, *p* = 1.0
SaO_2_ nadir (%)	84.9 (2.6)	82.3–87.8	84.4 (5.6)	76.3–90.4	*S* = 6.5, *p* = 0.79
SaO_2_ baseline (%)	94.9 (1.2)	93.3–96.2	94.8 (1.3)	93.3–96.0	*S* = 2.5, *p* = 0.79

Mean, SD, and range are shown for each time point. *p < 0.05.

## Discussion

We developed a protocol for IMT for sedentary adults with untreated OSA. We found that a 1-week practice period at 50% of maximum inspiratory resistance followed by 12 weeks at 65% with single daily practice sessions for a total of 13 weeks was achievable, manageable, and resulted in an average of 15–20% inspiratory muscle strength improvement. This was a significant increase, despite the small sample size. Target resistance and strength index data varied greatly between weeks in some patients. While the target resistance was fixed and only attempts counted that exceeded the target, any observed variation stemmed from patients taking stronger than necessary inspirations. We further established that initial IMT training should be supplemented with online and other digital resources and the opportunity to ask the research team members questions directly, especially during the first 2 weeks of training. We found increases in subjective daytime sleepiness, sleep quality, AHI, and RDI. While these effects were not significant, they provide an effect size for power calculations.

Our initial intention of participants performing two IMT sessions a day was not well received by our sample. Participants raised concerns about their ability to complete two sessions a day. The single daily session, however, did not elicit concerns and we observed 100% adherence across the 13 weeks, which was one of our major goals. During these 13 weeks, each participant completed a total of 91 IMT sessions involving 30 breaths each. This high adherence was potentially influenced by the initial rapport between participants and research team members, as well as the weekly phone contact. No participant reported logistical or motivation difficulties in performing the daily IMT practice, therefore we suggest that adherence may be increased in a clinical trial after participants are successfully introduced to IMT.

With regards to inspiratory muscle strength, our results suggest that ceiling or plateau effects in strength index occur after 6 weeks of training in some participants, consistent with previous published IMT studies. However, even in our small sample, there was variability in baseline performance capacity. One of our participants (Peppard et al., 2013) demonstrated a higher baseline capacity and continued to increase until 13 weeks. It is therefore possible that there is greater individual variability in both baseline performance, practice effects, and end results, including ceiling effects. The results from our sleep measurements demonstrate sharp improvements for participant 1 and less pronounced changes for the other four participants. However, only participant 1 had a greater than mild OSA severity. The subjective sleep quality rating showed improvements for all participants with a minimum of a 2-point improvement on a scale from 0 to 10.

While most existing IMT studies consist of 6 weeks of training and symptom-tracking (HajGhanbari et al., 2013), our 12-plus-1 week intervention was able to better detect potential ceiling effects in the data tracked daily or weekly. For instance, our results show the largest increases in average target resistance across the group from the practice period until the end of week 2. The slope then flattened but continued to increase in minor increments until the end of the intervention period. If we had only focused on the first 6 weeks, it would be unclear whether and with what increments the target resistance continued to increase or whether it remained at a plateau. Additionally, the extended intervention period allowed us to track and evaluate individual performance over time. Participant number 3 reached a target resistance of 57% after week 3 and was unable to increase the resistance for the rest of the intervention, while participant number 1 continued to increase the percentage in small steps to the end with 75% being the highest final resistance of the group. In terms of subjective experience, all participants reported positive changes, so one question that can be addressed in a clinical trial using a low-resistance control group, is whether any symptom improvements will be related to the mere daily practice (i.e., a placebo effect), or whether it was specific to the inspiratory strength gains. Participants reported appreciating the 1-week practice period at a moderate target resistance (50%). While this practice week may have induced a practice effect, the strength gains were likely due to the familiarity with the technique rather than muscle gain. We conclude that our adapted IMT protocol is suitable for testing whether IMT might be a beneficial alternative or complementary treatment to CPAP in sedentary people with untreated OSA. Using these modifications, we intend to perform a trial in which we randomize untreated OSA patients to active vs. low-resistance intervention arms, and investigate the effects of IMT on respiratory performance, sleep, and cardiovascular symptoms.

## Data Availability Statement

The raw data supporting the conclusions of this article will be made available by the authors, without undue reservation.

## Ethics Statement

The studies involving human participants were reviewed and approved by the University of California, Los Angeles Institutional Review Board (UCLA IRB #17-000918). The patients/participants provided their written informed consent to participate in this study.

## Author Contributions

PM conceived the idea for the pilot and trial and was the principal investigator. RA helped with the initial design, served as the medical professional and diagnosed participants. BK-S visualized and summarized the data and wrote the manuscript. AA and FM coordinated the study and gathered the data. EA contributed to the writing of the manuscript. RA served as the medical professional and diagnosed participants. All authors reviewed the manuscript.

## Conflict of Interest

The authors declare that the research was conducted in the absence of any commercial or financial relationships that could be construed as a potential conflict of interest.

## Publisher’s Note

All claims expressed in this article are solely those of the authors and do not necessarily represent those of their affiliated organizations, or those of the publisher, the editors and the reviewers. Any product that may be evaluated in this article, or claim that may be made by its manufacturer, is not guaranteed or endorsed by the publisher.
